# Reflections on Cerebellar Neuropathology in Classical Scrapie

**DOI:** 10.3390/biom11050649

**Published:** 2021-04-28

**Authors:** Adolfo Toledano-Díaz, María Isabel Álvarez, Jose-Julio Rodríguez, Juan Jose Badiola, Marta Monzón, Adolfo Toledano

**Affiliations:** 1 Departamento de Reproducción, INIA, 28040 Madrid, Spain; toledano@inia.es; 2 Instituto Cajal, CSIC, 28002 Madrid, Spain; miavmasueco@hotmail.com; 3 Biocruces Instituto de Investigación Sanitaria, IKERBASQUE, Basque Foundation for Science, Departamento de Neurociencias, Facultad de Medicina, Universidad del País Vasco-UPV, 48903 Baracaldo, Spain; j.rodriguez-arellano@ikerbasque.org; 4 Centro de Investigación en Encefalopatías y Enfermedades Transmisibles Emergentes—Universidad de Zaragoza, 50013 Zaragoza, Spain; badiola@unizar.es (J.J.B.); mmonzon@unizar.es (M.M.)

**Keywords:** classical natural scrapie, cerebellum, Purkinje cells, calbindin immunoreactivity, calretinin immunoreactivity, astrogliosis, microgliosis, spongiosis, abnormal PrP deposits/deposition

## Abstract

In this review, the most important neuropathological changes found in the cerebella of sheep affected by classical natural scrapie are discussed. This disease is the oldest known of a group of unconventional “infections” caused by toxic prions of different origins. Scrapie is currently considered a “transmissible spongiform encephalopathy” (due to its neuropathological characteristics and its transmission), which is the paradigm of prion pathologies as well as many encephalopathies (prion-like) that present aberrant deposits of insoluble protein with neurotoxic effects due to errors in their catabolization (“misfolding protein diseases”). The study of this disease is, therefore, of great relevance. Our work data from the authors’ previous publications as well as other research in the field. The four most important types of neuropathological changes are neuron abnormalities and loss, neurogliosis, tissue vacuolization (spongiosis) and pathological or abnormal prion protein (PrP) deposits/deposition. These findings were analyzed and compared to other neuropathologies. Various aspects related to the presentation and progression of the disease, the involution of different neuronal types, the neuroglial responses and the appearance of abnormal PrP deposits are discussed. The most important points of controversy in scrapie neuropathology are presented.

## 1. Introduction

Classical scrapie is a naturally transmissible spongiform neurodegenerative disease that originally affected sheep, goats and mouflons and that has been observed for several centuries [1,2]. The transfection of other animals has been performed in laboratory experiments designed to study the disease in greater depth [3,4,5,6]. It is a “strange” disease that cannot be classified as a classical bacterial or viral infectious disease and does not follow the pathogenic patterns identified by infectious or neurodegenerative disease research.

These spongiopathies in animals and in humans showed that one of the most affected regions was the cerebellum [7], which has generally shown resistance to degeneration/involution in many other diseases of the central nervous system (CNS) [8,9]. It has been demonstrated that the greatest alterations apply to both neurons and glial cells of the cerebellum [10,11,12].

Here, we collect and analyze the main data on cerebellum involution in classical scrapie of sheep to understand the neuropathology and clinical–pathological course of the disease in a unique natural model, which does not present the limitations observed in experimental research in the laboratory.

The special morpho-histochemical (neuronal and neuroglial) characteristics and the neuropathological manifestations (vesicular and toxic prion protein (PrP) accumulation) of the cerebellum will be analyzed to better understand these prion pathologies and identify possible therapeutic targets.

## 2. Historical Background

### 2.1. Cerebellum

The cerebellum is a very well-defined anatomical region of the CNS. It seems to be “isolated” from the rest of the brain and is connected by three pairs of peduncles. Its cellular structure and cytoarchitecture were among the first to be studied and described at the beginning of the neuroscience era. Consider the pioneering studies of Purkinje (see Vozeh, 2015) [13] and Ramón and Cajal [14,15,16] and his neurohistological school. The study of the cerebellum was key to establishing Cajal’s theories on the structure and function of the nervous system and describing the specific involvement of glial cells in the cytoarchitecture of each region of the nervous system [16,17,18,19,20,21]. Structurally, the cerebellum is composed of a series of deep cerebellar nuclei and a cerebellar cortex. The deep cerebellar nuclei receive information from the rest of the brain and produce responses from the cerebellum to different areas of the brain. The cerebellar cortex is a sophisticated regulatory (mainly inhibitory) structure of the deep cerebellar nuclei, and Cajal [16] indicated that it is structured with an outer layer or molecular layer and an inner layer with millions of interneurons, i.e., the “granule cells” that are excitatory for Purkinje cells (PCs), which are the main cells of the cortex located in the deeper area of the molecular layer. Despite Cajal’s original description, the presence of a third layer known as the PC layer is accepted. PC dendrites expand in the molecular layer, and their axons reach the deep cerebellar nuclei. The cortex receives information from the rest of the brain through two types of fibers: climbing fibers, which make synapses on the dendrites of the PC, and mossy fibers, which activate granule cells. There are many other types of interneurons in the cortex (stellate, basket, Golgi, Lugaro, monopolar or brush cells) that also regulate PC responses.

Transcendental studies for the development of neuroscience have been carried out in the cerebellum, i.e., “The cerebellum as a neuronal machine” (Eccles et al., 1967) [22], which is an essential book in neurophysiology as well as research on Bergmann glia, which is one of the first steps towards understanding the active role of astroglia in interneuronal synapses [23]. However, current cerebellum studies constitute well below 8% of the publications on the CNS. Thus, research on scrapie and animal and human prionopathies may be keeping publications on the cerebellum “alive”.

Cerebellum research has historically related this region of the CNS to movement. However, for more than twenty years, the cerebellum has been shown to contribute to the realization of certain higher cognitive functions in various types of memories (visual, verbal and spatial), prescriptive attention, learning, language and executive functions [24]. This is due to the extensive connections that the cerebellum has with the midbrain and diencephalic and telencephalic centers (especially in humans), although they have not been widely studied in other mammals [24,25,26]. The cerebellum is positioned as an “interface” between motor behavior and cognitive functions and as a supervisor of the performance of higher brain functions (“performance monitoring”) [27,28]. The cerebellum is currently considered to be important in the control of fear [29], social behavior [30], addiction [31] and human pathologies, such as autism [32] or non-motor language disorders [33]. The most frequent pathologies of the cerebellum in veterinary or human clinics are still cerebellar tremors, ataxias and dystonias that occur with age or in different diseases [34]. However, the cerebellum likely affects the behavior of all mammals.

The cerebellum presents a series of characteristics that make it “different” from other regions of the CNS. Among them, we want to highlight the following: (1) the large number of cells it contains; (2) the apparent simplicity of its basic morphofunctional structure; and (3) its resistance to involution [35,36,37,38]. Most studies on these subjects have been performed on the human cerebellum. The human cerebellum contains approximately 85 billion neurons [39], which is equivalent to approximately half of those contained in the cerebral cortex; however, it is assumed that not all of these neurons are functioning simultaneously [40]. Within this apparent histological simplicity, specific biomolecular subsets of the cells or morphofunctional modules or subregions within the cerebellum may be different [36,41]. The study of normal and pathological aging (Alzheimer’s disease, etc.) and of motor and non-motor pathologies of the cerebellum has revealed quite a few differences compared to other regions of the CNS when they regress or degenerate, although their characteristics are not well studied or defined [36,38,41,42].

### 2.2. Scrapie as Natural Prion Disease Model

Scrapie is an infectious disease of sheep that has been documented since 1700 [1], and it has been assigned various names throughout Europe. Its existence was later verified in goats [43]. Its origins may lie in Merino sheep in Spain, and its existence has been known since the 16th century (“trembling sheep”, “Tembladera”). The name scrapie refers to the lumbar itching of infected animals. A very interesting story about scrapie was published by Liberski (2012) [1], and it includes the research of two Nobel laureates (Gajdusek and Prusiner) as well as prestigious scientists (doctors, microbiologists, epidemiologists, biologists, etc.) that have been shaping what we know today about prion diseases. A considerable amount of time has passed since the knowledge of “rare” diseases of sheep shifted to a consideration of “spongiform encephalopathies” to “transmissible spongiform encephalopathies” (TSE) or “prion” diseases, and many scientific controversies have been observed. However, we now accept that prion pathologies are a “normal” group of neurodegenerative diseases, although they can contradict many of our principles on the DNA–RNA–protein chain that we consider basic in biology. Their pathogenic mechanisms (many of them in constant revision) have been recognized in many other neurodegenerative diseases (Alzheimer’s disease, Parkinson’s disease, tauopathies, etc.); thus, the term “prion and prion-like” is now used in many studies when referring to neurogenerative diseases. The common element among these diseases is the formation of non-degradable beta-protein aggregates (“misfolded proteins”) that are toxic to neurons (without discarding that soluble oligomers may mediate toxicity), which acts as the trigger for neurodegeneration. “Misfolding protein diseases” are a current priority in neuroscience research.

Ataxia and incoordination of movements as well as lumbar itching (scrapie) are the major symptoms of scrapie [44]. Therefore, studies on alterations in the cerebellum have been carried out with special interest. Prion diseases show that in mammal species other than sheep and goats (cows, dears and feline), including humans [1,12,45], the CNS shows considerable degeneration and the cerebellum is always affected.

**Figure 1:**
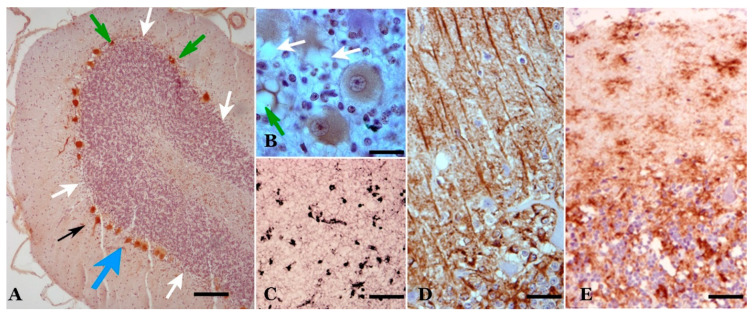
Main neuropathological alterations in classical scrapie. (**A**) Neuronal changes and loss. In these cerebellar folia, Purkinje neurons of different morphologies are observed: normal, hypertrophic (black arrow), atrophic (green arrows) and dystrophic (blue arrow). Likewise, areas exist where neurons have been lost (white arrows). Calbindin immunostaining plus hematoxylin contrast. (**B**) “Spongiform” (vacuolar) structures. Vesicles of different types are seen both in the neuropil (green arrow) and in the cytoplasm of neurons (white arrows). Calbindin immunostaining plus hematoxylin contrast. (**C**,**D**) Neuroglial reactivity. In (**C**), microgliosis is shown (proliferation of different types of microglia—rounded or branched). LN3 immunostaining plus Ni contrast. In (**D**), astrogliosis (hypertrophy, hyperplasia and increased number of gliofibrils) is shown. GFAP immunostaining plus hematoxylin contrast. (**D**) Abnormal prion protein (PrP) deposits/deposition. Various types of accumulations in the neuropil associated with cells, both in the form of dispersed or coalescent granulations and in a star-like shape. Abnormal PrP immunostaining plus hematoxylin contrast. (**E**) Abnormal PrP immunostaining plus hematoxylin contrast. (Bar: A = 125 µm; B = 20 µm; C = 125 µm; D = 30 µm; E = 35 µm).

Both classical scrapie and prion-like diseases induce neurodegeneration, with abundant neuropathological manifestations observable at the level of light and electron microscopy. Four important groups of alterations can be considered: (1) neuronal changes, (2) “spongiform” vesicular (or vacuolar) formations, (3) neuroglial alterations and (4) aberrant prion protein (PrP) deposits/deposition [10,11,12,44,45,46,47] (Figure 1).

Therefore, here, we highlight various factors which are extremely relevant. Firstly, histopathological investigation of the cerebellum has been crucial for the development of neuroscience. Secondly, we know little about the function of the cerebellum in terms of global mental and behavioral CNS function, both in humans and in other animal species. Thirdly, the cerebellum appears to be especially resistant to neurodegenerative processes, and fourthly, the cerebellum seems to be very sensitive in prion diseases. Lastly, an attempt is currently being made to group prion and non-prion neurodegenerative diseases, such as Alzheimer’s disease (so-called “prion and prion-like diseases”), based on the assumption of common etiopathogenic conditions, but there is great discrepancy regarding cerebellar neuropathology.

In this review, we aim to highlight the alterations studied in the cerebellum of sheep suffering from classical scrapie (Table 1) and their correlations observed in other mammals, including humans, to try to understand the neuropathology of all prion-like diseases.

## 3. Neuropathologic Alterations of the Cerebellum in Classical Scrapie

In all studies on scrapie, neuronal alterations have been noted, such as decreases in the various neuronal types of the cerebellum as well as glial alterations and other neuropathological changes (Figure 1). Although it is undeniable that there is neuronal loss, as has been described in other prion pathologies, we think that the dysfunctions seen in infected animals are more important than neuronal loss suggests. The neuronal alterations vary as the disease progresses, as we have summarized in some of our previous studies [10,11,48,49,50]. We will now analyze, in more detail, the four neuropathological characteristics presented in our studies.

### 3.1. Neuronal Alterations

**Figure 2:**
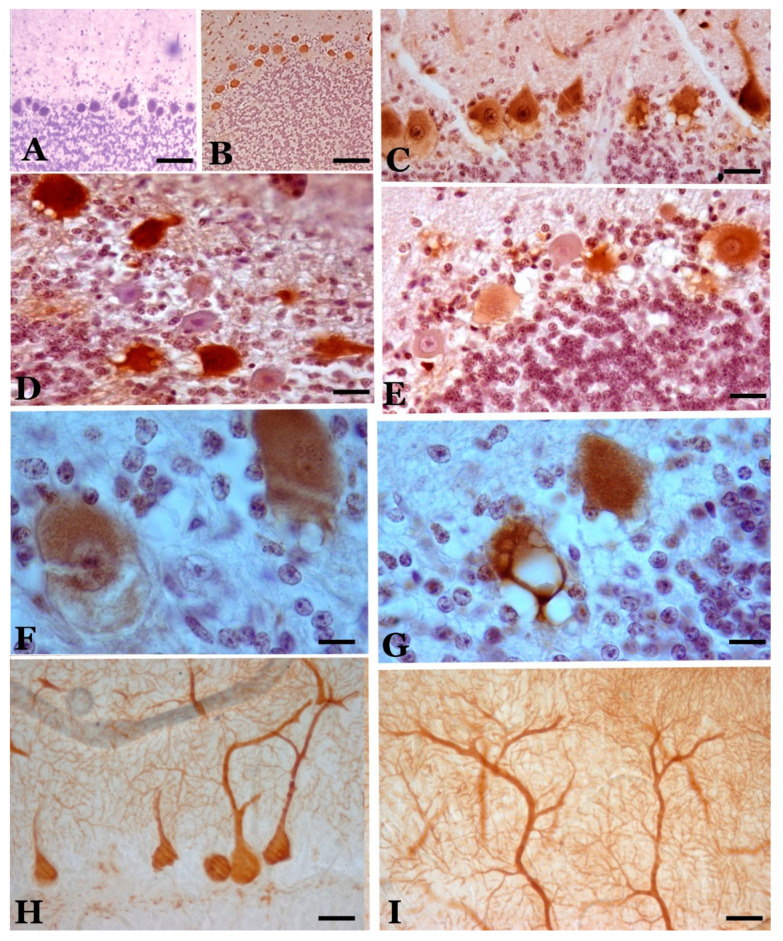
Calbindin-immunopositive Purkinje cells in control (**A**) and scrapie-affected sheep (**B**–**I**). (**B**) Cells of normal appearance in a case of preclinical scrapie, with a similar pattern to the control cases. (**C**) Cells of different appearance (normal, hypertrophic, dystrophic with rarefaction of their cytoplasm) in a case of clinical scrapie. (**D**,**E**) Cells of very different immunoreactivity in two cases of terminal scrapie. (**F**) Cytoplasmic rarefaction of the basal cytoplasm in an immunopositive cell of clinical scrapie. (**G**) Intense vacuolization in a positive cell. (**H**,**I**) Hypertrophic/hyperreactive cells. In (**H**), the hypertrophy of the initial dendrites is shown, and in (**I**), the hypertrophy of the distal dendrites that reach the pia mater. (**A**–**F**), hematoxylin contrast; (**H**,**I**), without contrast. (Bar: A, B = 125 µm; C–E = 25 µm; F, G = 20 µm; H,I = 30 µm).

**Figure 3:**
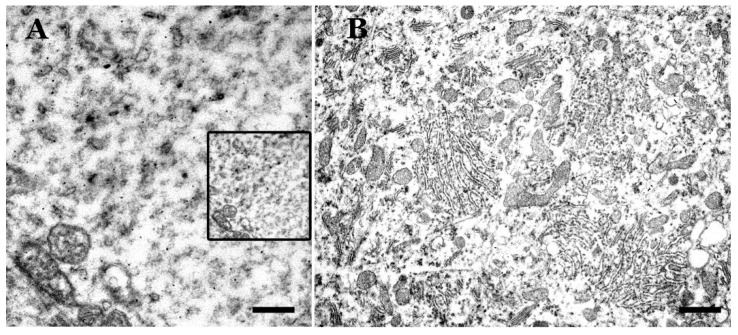
Electron microscopy images of the basal region of non-normal Purkinje neurons. Scrapie sheep in clinical phase. (**A**) Dystrophic cell with cytoplasmic rarefaction (dissolution/loss of subcellular organelles). Not defined debris are observed. Tubulo-vesicular formations appear (more notable in the upper left part of the image) as well as other electrodense microvesicles. Insert: magnification similar to (**B**) for comparison. (**B**) Hypertrophic cell (diameter, 42 µm) with a great abundance of subcellular organelles, although rough endoplasmic reticulum (RER) is more disorganized. Selected cytoplasmic regions are similar of those control neurons, but others resemble what is seen in dystrophic neurons (Bar: A = 0.5 µm; B = 3 µm).

**Figure 4:**
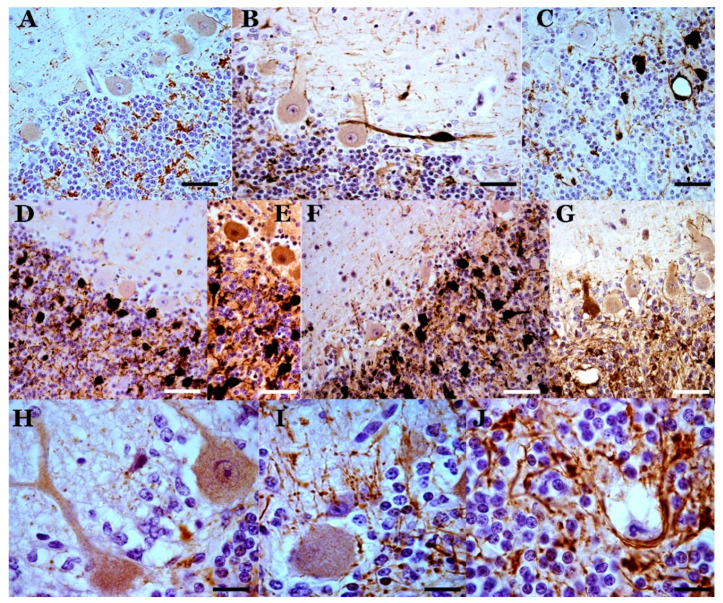
Calretinin-immunopositive cells in the cerebellar cortex of scrapie sheep (hematoxylin contrast). (**A**–**C**) Lobe VI (neocerebellum). In A, a slight reaction in Purkinje cells (PCs) and a greater intensity in some Golgi and brush cells of the granule cell layer as well as positivity in some parallel fibers are observed in a case of preclinical scrapie, a pattern similar to that shown by healthy controls. In (**B**), a case of clinical scrapie, the reaction pattern is maintained, although the intensity of the reaction is greater in some Purkinje neurons and cells of the granule cell layer (in the image, a Lugaro cell). In (**C**), a case of terminal scrapie, a very intense reaction is seen in hypertrophic Golgi cells of the granule cell layer (a vacuole is surrounded by its dendrites). The number of immunopositive Purkinje cells appears to decrease. (**D**–**G**) Lobe X (archicerebellum). In (**D**) and (**E**), a great diversity of reactions is observed in Purkinje cells (from negative to slightly positive in (**D**) to strongly positive in (**E**) as well as an intense reactivity in Golgi and brush cells of the granule cell layer. (The cell density of these cell types is 3–5 times higher than those observed in the neocerebellum). The reaction pattern is the same as that shown in control cases. In terminal cases (**F**)**,** it seems to increase the reactivity in Purkinje cells (some of them are very hyperreactive) and decrease the intensity of the reaction in cells of the granule cell layer. (**G**) Detail of hyperreactive hypertrophic Purkinje cells in a case of clinical scrapie. (**H**) Hypertrophic/hyperreactive PCs in clinical phase. (**I**) Thin calretinin-positive varicose fibers that are prolongations of astrocytes of the granule cell layer to the molecular layer (“Weigert fibers”), observable only in clinical and terminal cases. (**J**) Detail of the elongation of monopolar dendrites and axons of brush cells that form loops in their path between grains and vesicles. Clinical scrapie case. (Bar: A = 40 µm; B = 25 µm; C–G = 40 µm; H–J = 20 µm).

We do not consider the decreases in cell density in the sheep breed that we have studied (*Rasa Aragonesa*) significant when faced with senile involution, especially in preclinical or clinical phases. However, the histochemical changes of many neurons that can be the basis of the dysfunctions of the cerebellar circuits were significant [10,11,12]. It is important to note that the changes are different in the different cerebellar lobes and circumscribed areas in different lobes because they show important patterns (hyper- and hypo-reactivity of various markers and hypertrophy or atrophy of neurons) (Figure 2, Figure 3 and Figure 4). This could be more related to special “modules” or “functional circuits”, which are not yet well defined, within the apparent histological uniformity of the cerebellum. Moreover, individual responses of neurons in a specific subtype are always observed (Figure 2, Figure 3 and Figure 4). Ongoing investigations are attempting to differentiate the changes in different zebrine-positive or -negative strips and other possible functional zones, but have not yet yielded positive results, which have been observed in atypical scrapie. Individual responses are a possibility to be studied for theorical and practical considerations.

The total number of PC decreased by 13.3 ± 5.1%, and the granule cell layer section area decreased by 14.7 ± 7.1% [11]. However, in selected regions, a dramatic reduction in the PC density (>25%) and/or section area of the granule cell layer (>25%) was observed, with more involuted folia and a large reduction in immunopositive cell density. This pattern is most pronounced in the terminal phase of the disease [11].

Studies with markers of calcium-binding proteins (calbindin, parvalbumin and calretinin) have shown a great diversity of neuronal reactions in scrapie [51] (Figure 2 and Figure 4). Many authors have generically assigned the high expression of these proteins in the cerebellum as one of the causes of the resistance of this CNS region to involution/degeneration [36]. However, our studies have shown that the expression of these proteins in different neurons of the cerebellum seems to be more related to the functions of certain neuronal groups than to a general pattern of generalized response to prion infection [11].

Calbindin (CB) has been a very reliable marker for Purkinje neurons in sheep. Virtually 100% of the neurons in the young controls were immunopositive for calbindin [11,52,53]. During the pathogenic process of natural scrapie, a small decrease in neuronal density is observed (Figure 1 and Figure 2), and it is only slightly lower than that observed in senile animals. However, morphological alterations (presence of atrophic, dystrophic and hypertrophic cells—Figure 1, Figure 2 and Figure 3) indicate important neuropathological changes, which has been confirmed by studies at the electron microscopy (EM) level that show intracytoplasmic alterations that may be indicative of apoptosis or neuronal necrosis and alterations in Purkinje dendrites (and spines) and in some axons. Furthermore, a large number of intra- and extraneuronal vacuoles are observed (see below). Dystrophic neurites with autophagic vacuoles are considered, by some authors, to be the early stages of neuritic destruction [54].

Very intense calretinin (CR) immunopositivity in elements of all existing types of neurons and fibers in the cerebellar cortex of control sheep has been observed [11,52], although regional differences are very important, with high densities of immunopositive cells seen in lobules X and IX, a high density seen in lobe VIII and a low or very low density seen in lobule VIIb (Figure 4). In general, the immunopositive cells were more concentrated in the upper half of the granule cell layer. The number and location of these cells depend on the species of mammal and are distinct in sheep. Given these large variations in CR-immunopositive neurons and fibers, the assessment of changes in scrapie is difficult in many cases. However, variable patterns of CR-immunopositive structures have been recorded in different cerebellar cortical areas, and they range from an intense reduction in certain types of immunopositive neurons (mainly PCs in many folia of lobe VI) or fibers (mainly in the molecular layer of some folia of lobes VI and VII) to strong CR immunoreactivity in normal, hypertrophic or atrophic cells of all types (mainly in lobe X) (Figure 4). We can assume that variable responses are produced in different lobes (and in different folia of them).

In the clinical and terminal phases, many Purkinje neurons and certain stellate and basket cells, Golgi cells and Lugaro cells present immunoreactivity for various markers of apoptosis, necrosis or oxidative stress (iNOS, nitrotyrosine, caspases, HSP, etc.) [55,56].

Subpopulations of GABAergic neurons seem to be very vulnerable in prion diseases [57], but there are very important differences in the responses of subtypes (in the cerebral or cerebellar cortex), including PC neurons. In experimental models of scrapie, early increases and late decreases in PC dendritic spines have been observed [58].

### 3.2. Neuroglial Alterations

**Figure 5:**
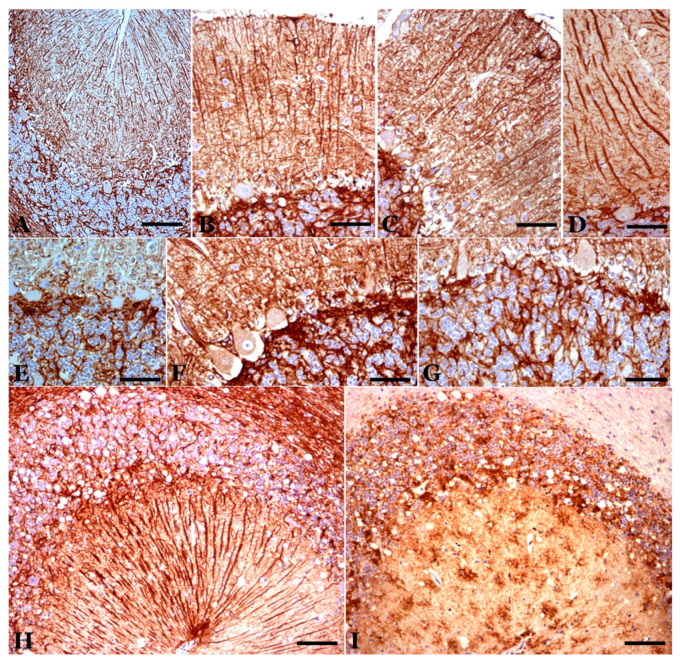
Astrogliosis in the cerebellar cortex of preclinical (**A**), clinical (**B**–**F**) and terminal (**G**) scrapie cases (GFAP immunostaining plus hematoxylin contrast). In the preclinical stage, the astroglial pattern is similar to that observed in controls. In the clinical stage, greater astrogliosis is observed, which can present different characteristics in different folia. In (**B**) and (**C**), there is hyperreactivity and hypertrophy in the astrocytes of the granular layer and in the Bergmann fibers of the molecular layer. In (**D**), great hypertrophy of these fibers is shown. (**E**) and (**F**) show greater astrogliosis in the basal area of the Purkinje layer. (**G**) Astrogliosis in a case of terminal scrapie. (**H**,**I**). Parallel GFAP-immunostained (**H**) and abnormal PrP-immunostained (**I**) sections, respectively. There is a coincidence of granular deposits of abnormal PrP deposits/deposition in the Purkinje layer and the granule cell layer, but the star-shaped deposits in the molecular layer do not correspond to GFAP-immunopositive stellate astrocytes in the molecular layer. (Bar: A = 50 µm; B–E = 40 µm; F,G = 25 µm; H,I = 50 µm).

**Astroglia**: GFAP immunostaining in healthy (control) sheep showed intense immunoreactivity in Bergman fibers and astrocytes in the molecular, PC and granule cell layers (Figure 5) and deep internal nuclei. A similar general pattern of glial distribution has been observed in preclinical scrapie-affected sheep (Figure 5). Nevertheless, quantitative differences existed concerning immunolabeling in the granular layer close to PC as well as in the white matter, which increased the reactivity compared to the controls. Moreover, some Bergmann fibers were hypertrophic/hyperactive, although their density in the molecular layer was not significantly different from that in young or senile controls. In animals at the clinical stage, the pattern of GFAP immunostaining largely differed from both control and preclinical animals primarily in terms of the morphology and intensity of the GFAP-immunopositive structures located in the molecular layer as well as in the upper part of the granular layer close to the PC layer (Figure 5). Thus, these structures appeared to include a perineuronal net of GFAP-immunostained processes as well as a high number of astroglial nuclei not associated with GFAP-immunostained processes (a sign of astroglial hyperplasia). In areas where Purkinje neuronal loss was more evident, GFAP-immunopositive elements decreased or even disappeared. CR immunoreactivity was observed in selected glial-like cells in scrapie-affected animals, mainly in cells near the PC layer (probably protoplasmic astroglial and Golgi epithelial cells) (Figure 5).

At the terminal stage of the disease, an intense increase in GFAP immunostaining was observed at the level of the PC layer (despite the maintenance or loss of the Purkinje neurons) as well as in some areas of the granule cell layer. Notably, glial processes of very high GFAP immunoreactivity were evidenced in all layers. These glial structures seemed to be conserved, even after neuronal death. Many hypertrophic/hyperactive (H/H) astrocytes, with H/H processes, were observed in all the layers. Note that the H/H elements occurred in diverse locations: subpial areas, Purkinje and granule cell layers and white matter. No outstanding differences were described for GFAP immunoreactivity, regardless of age, genotype or flock.

**Oligodendroglia**: Very few papers have been devoted to the study of oligodendroglia (including pro-oligodendrocytes or NG2 cells) in scrapie. Additional work on NG2 cells will be included in another chapter of this Special Issue.

**Microglia**: Most studies have shown that microglial cells increase in all layers of the cerebellar cortex (Figure 1) and the central cerebellar nuclei, with a moderate increase in the preclinical phase a more intense one in the terminal phase. In the preclinical phase, only star-like microglial cells were observed in areas near some PCs. In the clinical and terminal phases, large areas of all layers of the cerebellar cortex and deep cerebellar nuclei presented an increase in reactive microglial cells, including stellate forms and round phagocytic-shaped cells.

### 3.3. Spongiform Alterations

**Figure 6:**
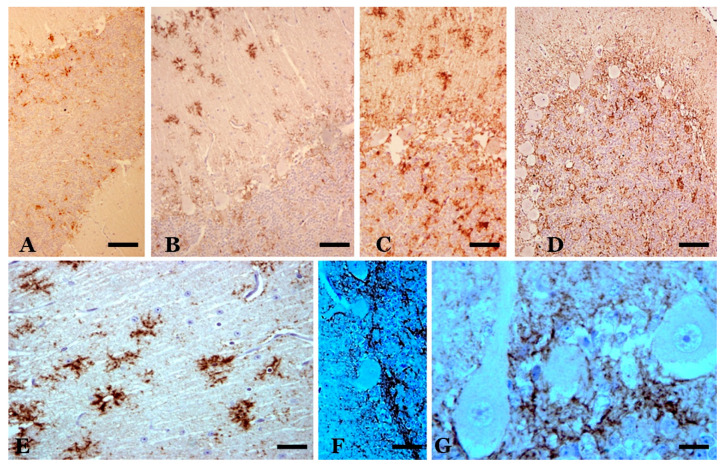
Abnormal PrP deposits/deposition (abnormal PrP immunoreaction—L42 1/500; Bio-Pharm, Darmstadt, Germany—plus hematoxylin contrast). (**A**) Deposits in a sheep in the preclinical phase of scrapie. Slight deposits are observed only in some folia in the layers of the cerebellar cortex. (**B**,**C**) Star-like deposits in the molecular layer (in **B**) and various types of deposits in all layers in two cases of sheep with infection in the clinical stage. (**D**) Diffuse granular deposits in all layers of a terminal-phase sheep case. Star-like deposits do not appear. (**E**–**G**) Details of prion deposits in clinical phase. In (**E**), star-like deposits are seen, which suggest association with non-GFAP-immunopositive astroglial cells. Hematoxylin-stained glial nuclei are seen in some of these deposits. In (**F**), a high degree of coalescence of the granular deposits is observed in the basal area of the Purkinje layer, similar to dense formations of astroglial processes in these areas (see Figure 4). In (**G**), a large accumulation of granular prion deposition is observed surrounding the areas of dystrophic Purkinje cells (with rarefied cytoplasm), but without the appearance of abnormal PrP immunoreaction in these cells. Abnormal PrP immunoreaction with hematoxylin contrast (in different concentrations). (Bar: A–D = 40 µm; E = 10 µm; F = 25 µm; G = 10 µm).

In the middle part of the granule cell layer, near the PC layer or in several areas of the molecular layer, vacuole-like structures were observed in scrapie-affected sheep. When these vesicles were studied in CB-, CR- or GFAP-immunostained sections, some of them were surrounded by CR-immunopositive neuronal structures (fibers and very dystrophic large cells), Purkinje neurons and/or glial-like processes (in both mild and intense immunopositivity) (Figure 2, Figure 4 and Figure 5). Sometimes, areas with these vacuolar structures lying close together with more intense abnormal PrP-immunoreactive deposits were observed in parallel sections immunostained for the prion protein (Figure 5 and Figure 6).

**Figure 7:**
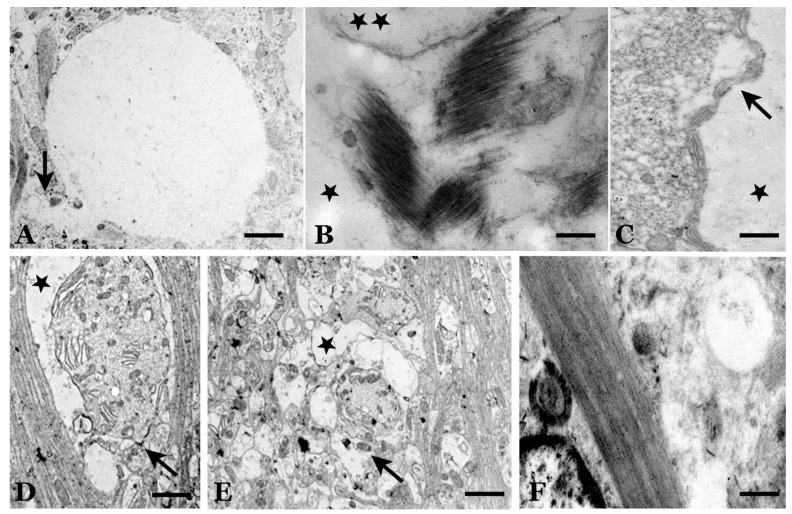
Electron microscopy images of vacuoles (“spongiosis”). (**A**) Large vacuole in a Purkinje cell without an apparent membrane. There seems to be an evagination (arrow, lower left area) towards the surface of the neuron. (**B**) Large (*) and small (**) vacuoles in the neuropil of the molecular layer surrounded by an astroglial process. Vacuole membranes are more or less apparent. (**C**) Large vacuole (*) in the neuropil of the molecular layer surrounded by a thin Purkinje cell dendrite (arrow), defined by accumulation of hypolemmal cisterns. (**B**) and (**C**). Section normal to parallel fibers. (**D**) Large vacuole surrounding a Purkinje dendrite that presents a large accumulation of hypolemmal cisterns but retains synaptic connections with climbing fiber terminals (arrow). (**E**) Various types of medium-sized vesicles surrounding Purkinje cell dendrites (*). Some vesicles lack cellular debris in their interior, but others retain cellular organelle debris (arrow). The latter may be vacuolized Bergmann fibers. (**F**) Hypertrophic astroglia cell with different types of vacuoles and intracellular dense bodies. (Bar: A,C,D = 3.5 µm; B and F = 1.5 µm).

**Figure 8:**
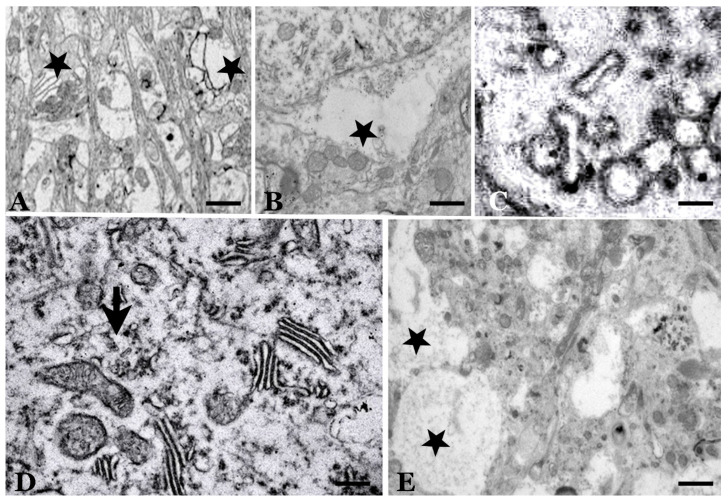
Electron microscopy images of vacuoles and subcellular bodies. (**A**) Dystrophic dendrites of stellate cells of the molecular layer (*), located between parallel fibers, showing autophagic vacuoles. (**B**) Intracellular vacuole without a defined membrane at an axon terminal of a basket cell on a Purkinje neuron (*). (**C**) “Tubulovesicular” formations with translucent content in association with electron-dense vesicles. (**D**) Cytoplasm of dystrophic Purkinje cells with loss of organoids, hypertrophic hypolemmal cisterns and microvesicles (arrow). (**E**) Purkinje atrophic cells surrounded by extraneuronal vesicles (*). They have a dense cytoplasm (“dark neurons”) with a large number of dense vesicular structures that are more or less complex. (Bar: A = 3.5 µm; B = 0.5 µm; C = 30 nm; D = 0.5 µm; E = 1 µm).

Inside neurons, particularly in PCs, vesicles of a large or small size were observed (Figure 1, Figure 2, Figure 4, Figure 5, Figure 6, Figure 7 and Figure 8).

### 3.4. Deposits of Aberrant Prion Protein

At the optical microscopy (OM) level, scrapie-affected sheep showed very intense pathological prion protein (PrPsc) immunoreactivity in lobules VIIb and X (Figure 6). The pattern of deposition was similar. PrPsc immunoreaction was observed in all cerebellar layers, thus revealing a non-homogeneous but constant presence of prion deposition. Small areas of variable size and shape with low immunoreactivity (fine and dispersed granules) were seen close together with other small- or middle-sized areas or “deposits”, with larger diameter granules (confluent in many instances) appearing in far greater numbers (Figure 6). The most intense immunoreaction was seen between the granule cells in the deeper region of the molecular layer and near the PC layer. The parallel sections processed for CB, CR or PrPsc immunodetection showed that the most heavily stained prion deposits were usually close to areas of low cell density between the granule cells, but not close to groups of cells showing CR immunoreactivity. CB-immunopositive PCs and CR unipolar brush cells of pathological appearance were observed close to the small areas of both very low and intense prion deposition. The parallel study of PrPsc- and GFAP-immunostained sections revealed variable correspondence between heavy prion protein deposits and hyperimmunoreactive glial cells (Figure 5). In different areas of greater density of GFAP-immunonegative glial bodies in the PC layer, intense PrPsc deposits were observed. In the clinical stage of the disease, the molecular layer shows a large number of star-like deposits, suggesting an association with astroglial cells, but GFAP-immunopositive astroglia of a similar morphology cannot be demonstrated in parallel sections (Figure 5).

When areas with large abnormal PrP deposits observed via light microscopy are studied at the level of electron microscopy, these large prion deposits cannot be confirmed. Only small aberrant formations associated with neuronal or glial membranes are seen, but they are not different from those observed in other areas not marked by large abnormal PrP deposits/deposition in OM studies.

## 4. Considerations for Studies on Cerebellar Neuropathological Alterations in Classical Scrapie

Since 1732 (first description of scrapie [1]), many neuropathological, biochemical (cellular and molecular) and genetic studies on natural scrapie in sheep and goats have been published. Post-mortem studies of infected animals are not conclusive, on many occasions, because of post-mortem degeneration as well as the difficulty of performing a sequential study of the progression of the disease over time, from the preclinical period to the terminal phase. The models used to analyze the pathogenetic course of this disease are diverse, and they have been developed from oral infection, the intracerebral injection of infecting prions into sheep and goats with different host prion genotypes, “infections” transmitted in different animal species (the rat, mouse—both “wild type” and genetically modified—hamster, etc.) and tissue sections or cell cultures. These studies have achieved important advances for clarifying the disease’s pathogenic course as well as the pathogenic mechanisms; however, great discrepancies are observed between biological study models and “classical” natural scrapie studies. These differences have been attributed to the different prion strains used in the studies, the transfection techniques and the time required by each model to produce involution of the neurons.

Neuropathological studies have focused especially on four aspects (Table 1): neuronal alterations and loss, vacuolization (intra- and extraneuronal) (spongiosis), neuroglial alterations (astrogliosis and microgliosis in particular) and abnormal PrP deposits/deposition. These characteristics are extremely important in the diagnosis of the disease, for differential diagnosis with other sheep diseases and for comparative studies of this pathology with other neurodegenerative diseases of mammals (including man), which many authors have grouped under the term “prion-like” diseases. This conceptual chapter includes all of the diseases, as mentioned above, that are dependent on poor normal protein catabolism and molecular changes of these proteins to a beta structure (“misfolding catabolic proteins”) that give rise to agglomeration of beta-proteins, which are not easily destructible and cause neuronal toxicity (neuronal dysfunction and/or death). Studies of the alterations and the neurodegenerative mechanisms underlying scrapie can be of great importance in advancing our understanding of human neurodegenerative diseases and identifying therapeutic targets for diseases that still have no treatment.

Of the many aspects studied in the cerebellum of animals affected by natural scrapie and simulated in experimental models, we will analyze the most important aspects.

### 4.1. Comparison of the Cerebellar Neuropathological Aspects of Classical Natural Scrapie with Other Neuropathological Alterations in Selected Brain Regions of Prion/Prion-Like Diseases

Table 2 shows the results of our small comparative study on the main neuropathological characteristics described in the cerebellar cortex in classic natural scrapie, in other regions of the brain in this disease and also in other prion diseases (atypical scrapie and human prionopathies), as well as in Alzheimer’s disease (AD, a paradigm of non-prion neurodegenerative disease within the prion-like diseases currently considered). It can be observed that the neuronal involutional alteration is important in prion diseases but not in AD, and that spongiosis is very constant in prionopathies but not in atypical scrapie. The deposit of toxic proteins is very important in prionopathies but not in AD. Astrogliosis and microgliosis are always present in all of these neurodegenerative diseases. It is shown that with regard to the cerebellum, there are clear neuropathological differences between both prion diseases and prion-like diseases. These differences have not yet been well explained and deserve a more exhaustive comparative study, since they could clarify the possible specific etiopathogenic mechanisms as well as reveal some specific therapeutic targets.

### 4.2. Sequence of the Temporal Appearance of Neuropathological Markers

The temporal sequence of the different neuropathological manifestations has been addressed in various studies. Some studies have pointed out that before neuropathological markers become patent, there are more subtle alterations that can only be detected by certain methods. Electrophysiological changes indicate functional alterations of the granule cells, with alterations in the neurotransmission of the parallel fibers in the upper areas of the molecular layer (including a decrease in these fibers) occurring before the appearance of other alterations [59]. An important conclusion is that the first deposits of infective prions (or the induction of the first pathological prions in the host) already seem to produce dysfunctional alterations in the cerebellum.

Studies on the progression of the disease show that several events are of great importance. First, the cerebellum does not present a uniform response to infection in all regions, because the different neuronal lines do not react in the same way, and certain regions of the cerebellum are more or less affected. Generally, more marked sensitivity is observed in Purkinje neurons than in Golgi, stellate, basket and granule neurons. However, within each neuronal lineage, there are individual responses, which may be morphologically identified by hypertrophy, dystrophy or atrophy or by hyper- or hypo-reactivity of some markers, especially calcium-binding proteins (calbindin and calretinin), or by more or less intense levels of necrosis or apoptosis.

Second, different areas show a greater or lesser degree of neuropathological involvement, which may be related to several factors. Despite the apparent homogeneous cellular architecture of the cerebellum (observed in its neuronal and glial composition of molecular, Purkinje and granular layers), it is composed of different lobes (in vermis and lateral masses) that present different areas, bands or regions with different gene expression (such as the zebrin protein), a greater density of some neuronal types (i.e., monopolar neurons and brush cells) or different types of fibers (mossy or climbing afferents). The prion infection pathways of the brain (including the cerebellum) can be utilized in several ways, with the afferent nerves being one of the most important; therefore, certain cerebellar areas may be infected earlier than others, and certain regions may be more intensely attacked by pathogenic prions. All of these differences would lead to cerebellar areas having greater or lesser involvement in natural scrapie.

### 4.3. Initiation and Progression of Cerebellar Neuropathology

The initiation of neuropathology in the cerebellum can be determined by the arrival of pathogenic prions to this region of the CNS. A number of studies have pointed out several pathways, which are not mutually exclusive [60]. The nerve pathway and the hematogenous pathway seem to be the most likely according to various studies, although another pathway through the cerebrospinal fluid is mediated by subpial astrocytes [12,61,62]. We speculate that climbing and mossy fibers may be a gateway to the cerebellar nuclei (through their collaterals) and the cerebellar cortex. Possible disruption of the blood–brain barrier does not appear to be very important, since prion infection usually originates in young animals.

In the absence of other analysis methods, multiple foci of pathogenicity appear to be possible, because focal areas of neuropathology have not been observed in preclinical phases. In models produced by cerebellar injection, it takes time for neuropathology and/or symptoms to manifest, which seems to indicate that there is an initial phase of dissemination of external pathogenic prions (with possible induction of pathogenic prions in the host in areas close to the initial foci) that are the substrate of global cerebellar infection.

To meet the theoretical postulates of prion pathology, the pathogenic prions newly formed by the host must come from cerebellar cells, which is the subject of several controversies. The cellular isoform of the prion protein (PrPc) displays predominantly neuronal localization [63,64], and neurons express prion protein mRNA [65]. Distinct types of synapses display differential PrPc, suggesting that they could influence variable patterns of CNS infection by prions [66]. It is moderately well accepted that the conversion of the PrPc into pathological prion protein (PrPsc) occurs in neurons. PrPsc deposits have been observed in some neurons during the development of clinical prion diseases [67]. Exosomes from these neurons can export both PrPc and PrPsc. Initially, the origin was mainly identified in Purkinje neurons.

However, several studies have indicated that astroglial cells are responsible for the high PrPsc accumulation in the cerebellum, which is either because these glial cells are capable of producing PrPc, which is transformed into PrPsc, or because they capture PrPc proteins that “transform” into PrPsc deposits. Several studies have demonstrated that in some breeds affected by natural scrapie, such as Suffolk sheep, the concentrations of PrPsc deposits associated with glia are even higher, especially in astrocytes [68,69,70]. The demonstration of cellular accumulation of PrPsc in astrocytes and the in situ hybridization of mRNA encoding this protein in astrocytes show that the scrapie agent replicates in astrocytes and induces the conversion of PrPc to PrPsc [68].

Accumulations of toxic prion proteins (abnormal PrP deposits/deposition) near neuronal or glial membranes do not necessarily indicate that their origin is the neuronal or glial cell that they appear to be associated with. After studying different types and variants of prion diseases in animals and humans and their experimental models, it is possible to describe different types of pathogenic prion formations as well as the involvement (primary or secondary) of neurons or glial cells.

We consider that there are three key elements for the production and accumulation of pathogenic prions: the source of the agent [69], the genotype corresponding to the host [70] and the different lines of neuronal and glial cells of each region of the CNS (in this case, the cerebellum). Differences in the relative affinity of different agents for different cell types may lead to differences in the cellular processing of PrPsc [71]. Therefore, different strains might preferentially target some cell populations, thus giving rise to a more prominent neuron-associated rather than glia-associated pattern of abnormal PrP deposits/deposition [72]. These differences might also explain the possible variations in tropism between the classical and atypical scrapie strains [73].

The progression of neuropathology within the cerebellum can vary from the areas primarily affected by the pathogenic prions. A very interesting study by Kim and colleagues [74] showed that the injection of pathogenic prions in the middle of a hemisected cerebellum in a murine scrapie model led to an intense alteration in the injected hemi-cerebellum and a low and/or delayed alteration in the non-injected hemi-cerebellum. This finding indicates that the disease presents a spatial spread. Although more detailed work has not been carried out in this field, modern studies on the production and diffusion of pathogenic/toxic molecules and neuroprotective elements by exosomes point out that this pathway may be one of the most important ones for the spread of pathogenic prions. This theory is fully integrated into the specific pathogenic theories on all prion and prion-like neurodegenerative diseases (Alzheimer’s disease, Parkinson’s disease, tauopathies, etc.).

### 4.4. Extra-and Intraneuronal Vacuolization: A Differential Feature Not Well Explained

The spongiosis (micro- and macrovacuolization) observed in this pathology, a differential characteristic of the group of transmissible spongiform diseases, has not been well elaborated thus far, although it is of great importance in diagnosis [75,76,77,78,79,80,81,82,83,84,85,86,87,88]. In studies at the level of optical and electron microscopy, a large number of vacuoles with a size of 0.3 to 20 microns have been evidenced, outside the neurons in the neuropil. At the same time, many neuronal somas and some of their dendrites show many vesicles of very different sizes, shapes and content. Some of these large vesicles, with translucent content, are similar to the vacuoles of the neuropil.

First, this pathological manifestation is not a pathognomonic sign of prion disease, since atypical scrapie (prion disease caused by other strains of prions) shows a null/minimal degree of spongiosis (Table 2). Similarly, whether the extraneuronal vacuolization in the neuropil is related to translucent vesicles observed in the soma and neuron dendrites has not been clarified. The neuronal autophagic vesicles/vacuoles could converge and then expand, and eventually, a vast area of the cytoplasm was transformed into a merging mass of autophagic vacuoles [75] (Figure 7).

#### 4.4.1. Extraneuronal Vacuoles

These alterations are not caused by the type of tissue (post-mortem) and its processing (fixation by immersion), because this neuropathological manifestation is not observed in other well-studied neurodegenerative processes (Alzheimer’s disease, Parkinson’s disease, etc.). Histological processing of the tissues (dehydration, fixation and staining) for observation at the level of optical (OM) or electron (EM) microscopy preserves the vesicular structure and does not show the internal contents (“empty vesicles”) in most cases [49]. This finding suggests that the “limiting membranes” or “diffuse limits” of these vacuoles are rigid enough to not collapse during histological processing. However, at the EM level, it is very difficult to observe a clear delimiting membrane of these vesicles in some instances, although a clear single or double membrane may be observed in other cases. This finding seems to indicate that we are not facing a single type of neuropathological manifestation, but rather several types of pathological changes.

Regarding the content, very few vacuoles show traces that can be stained by dyes or osmium tetroxide. Histochemical techniques have not shown any possible intravesicular components, especially PrPsc aggregates. These vacuoles represent a “mystery” to be clarified. Hydrophilic and hydrophobic materials have been investigated without any positive result. Though lipid materials are usually eliminated with tissue histological processing, as observed for lipid vesicles, other histological processes preserving the lipid content have been utilized, but no stained contents have been observed.

Several authors, as mentioned above, think that extrusions of autophagic vacuoles (or confluent vacuoles) can produce larger extraneuronal vacuoles (spongiosis). However, why not in other neurodegenerative diseases?

Extraneuronal vesicles can be surrounded by degenerative neuronal processes. Despite being so important for diagnosis, the role that these vesicles play in the pathogenesis of the disease has not been clarified. In our studies, we did not observe a clear relationship between extraneuronal vesicles and neuronal or glial abnormalities (Figure 7 and Figure 8). Many of the neuropil vacuolizations appear to be surrounded by glial processes, although others do not. Similarly, some of these vesicles seem to modify the morphology of the adjacent cells, while others only modify the path of the dendrites or axons, without showing any other type of adjacent cellular pathology.

Some studies on fixation by immersion in the cerebellum of healthy animals or animals with different neurodegenerative pathologies showed that many astroglial processes were more sensitive to cell destruction and that they showed “empty” spaces in the neuropil at the EM level. However, they were clearly identifiable as destroyed glial processes by their appearance—elongated spaces surrounding the neuronal dendritic processes. In older animals (rats and mice) or transgenic mice (Alzheimer’s disease, AD), the destruction of glial processes was significantly increased without showing any type of content, but without a preferential formation of vesicles.

#### 4.4.2. Intraneuronal Vesicles

Similar in size to large extraneuronal vacuoles in neuropil, large vesicles with a very low cell debris content or no apparent content are seen in some PCs (Figure 3). These vesicles seem to be produced by the destruction of the cytoplasm in the basal cytoplasmic area of these cells.

Many intraneuronal vesicles, especially those of a smaller size and with membrane debris, can be related to apoptotic or necrotic events that occur during the involution/degeneration of neurons (including hyperautophagy and excitotoxic stress [77]) as well as normal autophagy processes occurring in the cells as an adaptative mechanism. As defined by López-Pérez and colleagues [78], “autophagy is a fundamental cellular process involved in the turnover of long-lived proteins, protein complexes, cytoplasmic constituents and whole organelles through lysosomal degradation, in response to external and internal triggers”. The first role of autophagy is to clear cell debris and eliminate invading pathogens while simultaneously producing amino acids. A part of the neuronal cytoplasm was sequestrated within double or multiple membranes (phagophores) to be destroyed [75]. Autophagy modulation can control the dissemination of prions by interfering with their exosomal release [79]. Despite these pro-survival functions, autophagy can also mediate non-apoptotic cell death (“autophagic cell death”) [78,80]. The dual neuroprotective and neurotoxic roles of autophagia make it difficult to interpret what is happening in each phase of the disease and in each specific region of the cerebellum in scrapie-infected animals. Autophagy seems to be decreased in the late phases of the scrapie disease. In PCs, the autophagy machinery is still intact in less affected areas [80,81] and, probably, induces PrPsc clearance. These adaptive or pathological structures are not specific to scrapie and can be observed in senile animals or in other pathologies. However, in sheep scrapie, they appear more numerously at the end of the preclinical phase (with adaptative meaning) and in the terminal phase (with involutive meaning). Autophagic vacuoles and autophagosomes are a major part of dystrophic dendrites [75,76,77] (Figure 8). Most likely, all of these structures impair transport in dendrites and axons and the normal function of neurotransmission.

In an important number of EM studies on natural and experimental scrapie [3,49,82], a huge variety of vesicular pathological structures of lysosomal type or degenerative residual bodies were verified. These subcellular structures present a wide variety of sizes, shapes and content. Some of them present a clear single or double membrane, but in others, it is difficult to appreciate it. The content can be heterogeneous, including different types of more or less identifiable cellular debris, or, on the contrary, present as vesicles or empty spaces or as dense osmiophilic masses. Several of these formations are known by various names. The most frequently referenced are aggresomes, which are round electron-dense structures and not membrane-bound [81]; tubulovesicular structures (TVS, vesicular structures of higher electron density than synaptic vesicles, of approximately 27 nm in diameter within axonal terminal or dendrites) of unknown significance; electron-dense bodies; “whorls” or concentric arrays of membranes; debris of apoptotic cell nuclei, etc. [76,81,82,83,84,85,86,87]. It should also be noted that in normal sheep, many smooth endoplasmic reticulum formations are observed in the soma and dendrites of PCs (“spine apparatus”, hypolemmal cisterns), which can give rise to very extensive tubulovesicular formations of unknown significance, although they are not necessarily pathological.

### 4.5. Alterations and Neuronal Loss: Neuronal Specifity

Although significant neuronal loss has been reported in all studies on the cerebellum of animals with classical scrapie, different neuronal lines regress/degenerate in different ways during the progression of the disease. It should be noted that there are also regional differences of different neuronal types in different areas/regions of the cerebellum and individual differences in some elements of a neuronal type (PCs, monopolar cells, etc.) that manifest in morphological or histochemical changes in different areas, with more or less effects before neurotoxic prion attack.

Apart from neuronal loss, several significant specific aspects of neuronal dysfunction have been studied in greater depth:

(1) Alterations in neuronal synaptic regions. Studies of the localization of normal cellular prion molecules seem to indicate that they are homogeneously localized on all surfaces of neurons, including the soma, dendrites and axons. However, several studies have indicated that the greatest neuropathological alterations are observed in dendritic areas with high synaptic functions or in axons of these neurons. The initial accumulations of neurotoxic prions (external and/or induced in the host) could be focused on areas of high neurotransmission. The evolution of areas of high synapse concentration in scrapie has been reported in several experimental studies.
(2) In different neuronal types, especially in Purkinje neurons, various types of morphological alterations have been shown (as mentioned above), especially at the level of EM, and they have been interpreted in different ways over the years [76,81,82,83,84,85,86,87,88,89]. Some alterations (especially the so-called “tubulovesicular structures”) were primarily considered to be specific for prion diseases but were later “declassified” as pathognomonic markers of the disease [84,85,86,87]. Axons and dendrites of PCs have shown a large number of pathological structures with a very high variety of shapes and sizes. However, these structures have been observed in many other situations (aging and neurodegenerative diseases), and their interpretation is primarily hindered by the lack of a clear definition and a lack of information on the corresponding cellular or subcellular alterations. Some of these structures correspond to neoformations of the smooth or rough endoplasmic reticulum, while others correspond to lysosomal formations or accumulations of products and/or cytoplasmic areas of cell degradation. In different studies on experimental models of neurodegenerative diseases (especially transgenic mice), many of these structures have been observed; however, whether they are markers of specific neurotoxic changes or consequences of cell involution has not been clarified.

### 4.6. Neurogliosis: A Main Feature

Neurogliosis is considered a marker of prion disease, first as astrogliosis and then as microgliosis. Astrogliosis is inherent to prion disease [6,12,62,90,91,92,93]. GFAP hyperreactivity is manifested in the cerebellum, especially in the Bergmann fibers of the epithelial glia, as well as in different stellate astrocytes of the molecular layer and the granule cell layer. However, in studies on the density of Bergmann GFAP-immunopositive fibers, we did not find significant variations in the number of fibers, although an increase in astroglial-like nuclei without GFAP-immunopositive surrounding cytoplasm was observed in the Purkinje layer. Taking into account that astrogliosis includes both the hypertrophy/GFAP-hyperresponsiveness of glial cells and hyperplasia of the same (which is difficult to appreciate with histochemical techniques, since many newly formed glial cells are GFAP negative), it can be assumed that there is a great dynamic of the neoformation/destruction of glial cells in prion disease. Similarly, in all layers (molecular, Purkinje and granule cell layers), astroglial changes are seen, with some observable by GFAP staining while others are not. These astroglial alterations may be associated with induced phenotypic changes rather than constitutional astrocyte responses. Some of the changes are more evident at the molecular transition of Purkinje–granule cell layers.

Microgliosis in scrapie has been analyzed with more interest in recent years, perhaps related to the increased interest in the “neuroinflammation” process attributed to the genesis of neurodegenerative processes. Microglial activation has been identified in many studies in the cerebellum of wild-type scrapie-infected animals. In different studies, hyperplasia and hypertrophy of these cellular elements have been shown, although their importance in prion infection is, today, a controversial matter. Positive and negative effects have been pointed out [12,94,95,96,97,98,99]. In several studies, it has been shown that prion infection causes important neurotoxic effects that are absolutely deleterious when they occur along with a neuroglial neuroinflammatory reaction. Microglia seem to be critical in host defenses against prion diseases by removing prions [96]. The disease progression is faster when microglia are depleted [94]. Bone marrow-derived cells seem to “sense” early pathological changes in the brain during the incubation of scrapie and can engraft throughout the CNS in substantial numbers as new invasive microglia, concomitant with the deposition of abnormal PrP [100].

It is noteworthy that astrogliosis and microgliosis are common features of all neurodegenerative diseases (Table 2).

Modifications of oligodendroglial cells have been observed in some prion diseases [101], but they have not been studied in depth.

### 4.7. Abnormal Prion Protein Deposits: Diversity and Controversial Cell Association

Different abnormal PrP immunostaining patterns of deposits are a common situation In order in most prion diseases, and “amyloid” (PAS-positive) deposits are characteristic features in prion-like pathologies. Different types of abnormal PrP deposits have been observed, including patchy/perivacuolar, surrounding spongiform structures; diffuse/synaptic in the neuropil; perineuronal or plaque structures of different typology [12,62,102]. In the cerebellum, a wide manifestation of all of these types exists.

One of the main neuropathological markers of natural classical scrapie is the accumulation in the cerebellum of prion deposits. At the OM level, they can be found in all layers of the cerebellar cortex, especially in the granule cell layer; however, the deposits are difficult to identify at the EM level. Large “masses” of protein deposits at the level of light microscopy have no correlation with the deposits at the EM level, and this issue has never been clarified.

In classical scrapie, the OM protein masses are very variable, with some forming plaques similar to those seen in other prion pathologies; however, the incidence of granular components (more or less diffuse, with many of them being confluent) is similar to that of “diffuse” amyloids from other neuropathologies, such as Alzheimer’s disease.

At the level of EM, some tubulovesicular structures that were initially classified as “prion markers” are no longer considered scrapie markers.

The most important “masses” of prion accumulation tend to surround the granule cells and occupy the spaces of the mossy fibers, suggesting a zone of great neurotoxicity of the afferents to the cerebellum.

Prion protein deposits are mostly associated with neuronal or glial membranes and sometimes cause many problems of interpretation when studied. In the clinical phase of scrapie, star-shaped abnormal PrP deposits appear that do not correspond to immunopositive GFAP astrocytes in parallel sections. In other parallel sections immunostained to identify other cells (microglia, NG2+ and neurons), association with other neuronal types has not been clarified. Our results are consistent with a close relationship with astroglial cells, but what subtype of astroglial cells? This is what we will try to cover in the upcoming work mentioned above (Section 3.2; oligodendroglia).

The correlation between spongiosis, prion protein deposits and surviving neurons is controversial in all prion diseases [12,62,76,102].

## 5. Conclusions and Future Perspectives in the Research

In this review, we have tried to highlight several important facts in the study of prion diseases (transmissible spongiform encephalopathies) by focusing on natural “classical” scrapie in sheep and goats. This “strange” disease, which is very different from our concepts of infectious diseases, can provide theoretical and practical insights for new pathologies that not only genetically affect various animal species but can also be transmitted to humans. These insights have led to many questions about the neurodegenerative mechanisms of and the potential therapeutic action against this disease. In classical scrapie, the route of infection and the host response are important, because the formation of neurotoxic prions and the neuroprotective/neurodegenerative responses of the nervous tissue are essential for understanding and fighting this disease and other related pathologies. Neurodegeneration can reveal pathogenic pathways that can be therapeutic targets, and although adaptative and reactive neuronal responses in this natural disease are invalid in the long term, they can indicate therapeutic alternatives for this pathology and other neurodegenerative diseases.

Although many works have focused on natural classical scrapie and experimental scrapie, many issues regarding the pathogenesis and pathogenic course of the disease have not been clarified and need more research. Many cellular and molecular alterations described in the disease are poorly understood or interpreted speculatively, without a certain scientific basis. The involution of the different types of neurons (both dependent on their lineage and/or individually, dependent on their specific status) as well as the neuroglial reactions, which can be neurotoxic as well as neuroprotective, are not sufficiently clear in scrapie. Neurotoxic prion infection and host response “models” are very important research tools for understanding neurodegeneration mechanisms and developing therapeutic strategies, but they necessitate more in-depth study to solve problems and not increase theoretical controversies.

Additional investigations of the morphofunctional alterations of classical scrapie (vacuolization, neurodegeneration, gliosis and abnormal PrP deposits/deposition) are required to fully discover the specific neurotoxic mechanisms of these prion pathologies as well as other generic neurodegeneration mechanisms in brain pathologies.

Studies on the cerebellum and its pathological processes are of great relevance, since, in recent years, the importance of this region of the CNS in the control of many mental and behavioral mechanisms has been described (as highlighted in Section 2) but has not yet been well studied. Not only is the cerebellum a motion controller, but it is also integrated into other CNS organizer networks of higher brain processes. It merits further research into its normal situation and pathological involution.

## Figures and Tables

**Table 1:** Cerebellar neuropathological abnormalities in classical natural scrapie considered in this review (Section 3) and reflections on these studies on cerebellar neuropathological alterations in classical scrapie (Section 4).

Cerebellar Neuropathological Abnormalities in Classical Natural Scrapie	
Cerebellar Cortical Layers	Abnormalities
Molecular layer	Decrease in thickness
Purkinje Cell layer	Loss and morphohistochemical alterations of neurons
Granule cell layer	Decrease in thickness Loss of granule cells
**Neurons**	
PC neurons	Decrease in cell density Morphological changes: Existence of atrophic, dystrophic, hypertrophic cells Cytoplasmic rarefaction, vacuolation Histochemical changes in Calbindin and calretinin immunostaining
Lugaro, Golgi and brush cells	Small morphohistochemical changes (dystrophy)
Granule cells	Decrease in cell density
**Spongiosis (vacuolation/vacuolization)**	
	Intraneuronal vacuolization
	Extraneuronal vacuolization
**Neurogliosis**	
Astroglia	Astrogliosis Astroglial hyperplasia
Microglia	Microgliosis
**Abnormal PRP Deposits/Deposition**	
	Different types of immunodeposits Different association to cells
**Reflections on the Neuropathological Cerebellar Changes**	
Comparison with other neuropathological brain patterns in selected brain regions of prion/prion-like diseases	
Sequence of the temporal appearance of neuropathological markers	
Initiation and progression of neuropathology	
Extra- and intra-neuronal vacuolization: a differential feature not well explained	
Alterations and neuronal loss: neuronal specificity	
Neuroglioses: a main feature	
Abnormal prion protein deposits: diversity and controversial cell association	

**Table 2:** Main neuropathological characteristics described in the cerebellar cortex in natural scrapie in comparison with other regions of the brain in this disease and also in other prion diseases (atypical scrapie and human prionopathies), as well as in Alzheimer’s disease (AD).

Pathologies	Neuronal Loss	Spongiosis	Neurogliosis	Aberrant Protein Deposition
CLASSICAL NATURAL SCRAPIE				
Cerebellum	+/+++	+++	+++	+++ (*)
Frontal cortex	NE	+	+++	++ (*)
Obex	NE	+++	+++	+++ (*)
ATYPICAL SCRAPIE				
Cerebellum	+/+++	−	++	++ (*)
HUMAN PRION DISEASES				
Cerebellum	+/+++	++/+++	+++	+++ (*)
ALZHEIMER DISEASE				
Cerebellum	−/+	−	−/+	−/+ (**)
Frontal cortex and hippocampus	++/+++	−	++/+++	++/+++ (**)

− to +++ = level of intensity; NE = not studied; * abnormal PrP deposits/deposition; ** = beta amyloid deposits.
